# Progressive Liver Fibrosis in Non-Alcoholic Fatty Liver Disease

**DOI:** 10.3390/cells10123401

**Published:** 2021-12-02

**Authors:** Daryl Ramai, Antonio Facciorusso, Erika Vigandt, Bryan Schaf, Waleed Saadedeen, Aditya Chauhan, Sara di Nunzio, Aashni Shah, Luca Giacomelli, Rodolfo Sacco

**Affiliations:** 1 Division of Gastroenterology and Hepatology, University of Utah, Salt Lake City, UT 84132, USA; Daryl.Ramai@hsc.utah.edu; 2 Section of Gastroenterology, Department of Medical and Surgical Sciences, University of Foggia, 71122 Foggia, Italy; antonio.facciorusso@virgilio.it (A.F.); r.sacco@ao-pisa.toscana.it (R.S.); 3 Department of Internal Medicine, The Brooklyn Hospital Center, Brooklyn, NY 11201, USA; evigandt@tbh.org (E.V.); bschaf@tbh.org (B.S.); wSaadedeen@tbh.org (W.S.); achauhan@tbh.org (A.C.); 4 Polistudium s.r.l., 20135 Milano, Italy; sara.dinunzio@polistudium.it (S.d.N.); aashni.shah@polistudium.it (A.S.)

**Keywords:** non-alcoholic fatty liver disease, non-alcoholic steatohepatitis, hepatocellular cancer

## Abstract

Non-alcoholic steatohepatitis (NASH) is a chronic and progressive form of non-alcoholic fatty liver disease. Its global incidence is increasing and makes NASH an epidemic and a public health threat. Non-alcoholic fatty liver disease is associated with major morbidity and mortality, with a heavy burden on quality of life and liver transplant requirements. Due to repeated insults to the liver, patients are at risk for developing hepatocellular carcinoma. The progression of NASH was initially defined according to a two-hit model involving an initial development of steatosis, followed by a process of lipid peroxidation and inflammation. In contrast, current evidence proposes a “multi-hit” or “multi-parallel hit” model that includes multiple pathways promoting progressive fibrosis and oncogenesis. This model includes multiple cellular, genetic, immunological, metabolic, and endocrine pathways leading to hepatocellular carcinoma development, underscoring the complexity of this disease.

## 1. Introduction

Non-alcoholic fatty liver disease (NAFLD) is an umbrella term that involves a spectrum of liver pathology, including simple steatosis, steatohepatitis, fibrosis, and cirrhosis. Multifactorial influences of diet, host genetics, and the gut microflora contribute to progression through the various pathologies of NAFLD. These insults ultimately cause hepatocellular injury and cell death [1].

Non-alcoholic steatohepatitis (NASH) has been demonstrated to have a close association with obesity and obesity-related complications, such as type 2 diabetes (T2D) [2,3]. With the urbanization of the global population in addition to changes in dietary habits, the incidence and prevalence of obesity have been constantly rising. As a result, a spectrum of diseases associated with an increased body mass index (BMI) has also arisen, such as diabetes, metabolic syndrome, and also liver injury diseases, including NAFLD and non-alcoholic steatohepatitis (NASH). Consequently, NAFLD has become a growing epidemic that is associated with significant morbidity and mortality, placing a strain on the quality of life and liver transplant requirements.

In a study of autoptic reports, the prevalence of NASH in obese patients was 18.5% [2]. A systematic review and meta-analysis estimated a global prevalence of NASH to be 37.33% (95% CI: 24.70–50.02%) among patients with T2D [4]. Nonetheless, it is imperative to not ignore the impact of NASH in the non-obese population. The prevalence of NASH in lean patients was found to be 2.7% [4].

In the USA, the National Health and Nutrition Examination Survey III (NHANES III) evaluated approximately 30,000 persons aged 2 months and older from 1988 to 1994 and identified a prevalence rate of NAFLD that ranged between 2.8% and 30.2%, based on different diagnostic criteria, such as ultrasound and liver enzyme levels [5]. Using only the measurements of alanine aminotransferase (ALT) or aspartate transaminase (AST), a prevalence rate of 5.4% was observed [6]. Subsequent studies have shown the growing prevalence in the USA, with the NHANES I identifying a higher prevalence rate of 8.1% from 1999 to 2002, to 17.6% in 2011 based on a US Veteran’s study [7,8].

In the Asian Pacific region, a similar rising trend was observed, with the prevalence of NAFLD being 25.3% between 1999 and 2005 compared to a rate of 33.9% from 2012 to 2017 [9]. Globally, an average prevalence rate of 15% in 2005 and later 25% in 2010 based on regression analysis, with the greatest increase being observed in Asia and the Pacific, has been observed [4,10].

The Hispanic race has been found to have the highest prevalence rates, with individuals from Mexico having higher rates compared to those from the Dominican Republic and Puerto Rico. On the other hand, African–Americans have the lowest prevalence despite having higher rates of obesity, based on the 6-year NHANES III trial using AST or ALT in a population of 15,676 participants [6,11]. Genomic studies have identified the *PNPLA3* gene as being strongly associated with the discordance of prevalence between races and liver steatosis. Specifically, the 148M allele was more common within Hispanic populations, while the S453I allele was found to contribute to low fat content in the livers of African–American individuals [12].

The progression of NASH to hepatocellular carcinoma HCC was initially defined according to a two-hit model involving an initial development of steatosis, followed by a process of lipid peroxidation and inflammation. In contrast, current evidence proposes a "multi-hit" or "multi-parallel hit" model that includes multiple pathways promoting progressive fibrosis and oncogenesis. This model includes multiple cellular, genetic, immunological, metabolic, and endocrine pathways leading to HCC development, underscoring the complexity of this disease [13].

## 2. NAFLD Risk Factors

Many of the identified risk factors for NASH are related to metabolic syndrome, also known as insulin resistance syndrome, and its related health comorbidities. Metabolic syndrome is defined by the presence of three of the following five major components: central obesity, elevated fasting blood sugar, high triglycerides, hypertension, and low high-density lipoproteins [14]. With each feature of metabolic syndrome, the risk of hepatic steatosis increases exponentially. Furthermore, the diagnosis of the metabolic syndrome itself increases the probability that a patient will have progression to NASH rather than simple steatosis [15].

NAFLD and NASH can be seen in obese and non-obese patients; however, the majority of the disease is seen in the overweight population. NAFLD is more common in obese patients (74% in obese patients and 90% in morbidly obese patients) [16]. Interestingly, the distribution of fat might be more important than the total fat mass as a predictor of NASH. Cross-sectional studies reveal a strong correlation between visceral adiposity and the prevalence of NAFLD, as well as the extent of steatosis. The odds ratios for increasing liver inflammation and fibrosis were 2.4 (95% CI: 1.3–4.2) and 3.5 (95% CI: 1.7–7.1), respectfully, for every 1% increase in visceral fat [17]. Petta et al. utilized a Visceral Adiposity Index as a marker of adipose dysfunction. Adiponectin is a well-known hormone secreted by adipocytes and proven to be influential in regulating the metabolism of lipids and glucose. Visceral adiposity has an inverse relation with adiponectin levels seen in patients with NAFLD. Researchers found that higher Visceral Adiposity Index, defined by a cut-off greater than 2.1 (sensitivity 69%, specificity 70%), was correlated with more significant hepatic fibrosis (F2–F4) [18].

Diabetes itself is identified as an independent predictor of cirrhosis and liver-related deaths [19]. Among patients with T2D, 33–66% have NAFLD [20]. As per Pang et al., the hazard ratio for individuals with diabetes compared to those without is 1.49 (95% CI: 1.30–1.70) for liver cancer, 1.81 (95% CI: 1.57–2.09) for cirrhosis, and 1.76 (95% CI: 1.47–2.16) for NAFLD [21]. Biopsy studies showed that T2D was the strongest prognosticator of NASH and hepatic fibrosis progression [11]. An analysis of almost 2.5 million Canadian patients newly diagnosed with T2D over a 12-year period showed a two-fold increased risk of cirrhosis, liver failure, or liver transplant [22]. Inversely, multiple studies have demonstrated that NAFLD and NASH place patients at higher risk for the development of T2D, suggesting that T2D is not only a cause but also a consequence of NAFLD and NASH [22].

Additionally, pro-inflammatory dietary components have been demonstrated to have a role in promoting NAFLD. Studies have shown that it is the balance between n-6 and n-3 polyunsaturated fatty acids (PUFAs) in the blood and liver, which is closely associated with the severity of NAFLD [23]. n-6 PUFAs are classified as proinflammatory, whereas n-3 PUFAs have more anti-inflammatory properties. Thus, the ratio of n-6:n-3 PUFAs is noted to be directly proportional to the progression of NAFLD. Furthermore, oxidized linoleic acid metabolites are formed products of n-6 PUFA linoleic acid oxidation which have also been shown to be elevated in individuals diagnosed with NASH [13]. Decreasing visceral fats can decrease hepatic insulin resistance. Lifestyle modifications, including weight loss, exercise and diet, demonstrated effectiveness in considerable slowing and regression of inflammation and fibrosis in patients with NASH [24,25]. Lifestyle modifications are recommended as the primary treatment of patients with NASH. Studies show that losing 5–10% of weight can offer a significant improvement in NAFLD and NASH [26].

**Figure 1:**
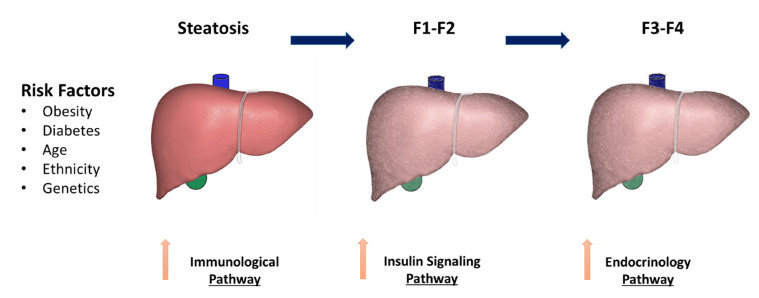
Risk factors and proposed mechanisms for non-alcohol steatosis (NASH) progressive liver fibrosis.

There are nonmodifiable risk factors, such as age, genetic composition, race and ethnicity, that have been attributed to influencing the progression of NAFLD to NASH (Figure 1). Age older than 45 or 50 years is associated with a higher likelihood of developing NASH, which is part of a multi-hit hypothesis [13]. A large multiethnic population-based study showed that the Hispanic population in the USA had a higher risk for NASH compared to that of European descent [27]. The study also showed that African–Americans were protected independently of diabetes and BMI [27]. Moreover, genome-wide scans and case–control studies have identified many different genetic variants that influence progressive liver disease, such as the *PNPLA3* gene, which will be discussed later in the manuscript [28].

## 3. Lipid Accumulation

Multiple mechanisms have been hypothesized to contribute to lipid accumulation in the liver. Free fatty acids have been attributed to account for about two-thirds of lipid deposition in the liver [29,30]. High-fat diets and lack of physical activity couple to promote the development of the metabolic syndrome. The excessive nutrients lead to adipose tissue accumulation, which can become dysfunctional due, in part, to the dysregulation of adipokines [31].

Insulin resistance is also associated with lipid accumulation and is defined as a state in which cells fail to react to the effects of insulin on glucose production. High levels of insulin can advance hepatic steatosis via impaired skeletal muscle and hepatic insulin signaling [32]. At the visceral adipose tissue, this impaired insulin effect leads to triglyceride breakdown and formation of free fatty acids. The liver then uptakes free fatty acids where it assembles as triglycerides [33].

Once the initiation of steatotic changes begins, oxidative stress, inflammation, and activation of stellate cells contribute to promoting the progression to NASH and early fibrosis. In patients with obesity and steatosis, the progression to fibrosis is accelerated by rapid weight loss during dieting, intestinal bypass surgery, surgical stress, alcohol intake, and T2DM, all of which increases free fatty acids in the liver [34].

Elevated levels of free fatty acids within the liver provides a source of oxidative stress by peroxisomal b-oxidation. This leads to the production of hydrogen peroxide. Highly reactive hydroxyl radicals are produced in the presence of iron. The release of these free radicals contributes to mitochondrial damage and the progression of liver fibrosis [35,36,37,38]. This supply of oxidative stress is needed for initiating enough lipid peroxidation to surmount normal cellular defense mechanisms and produce necroinflammation [39,40].

Furthermore, elevated biomarkers of oxidative stress and antioxidants have been implicated in the pathogenesis of NAFLD [41]. Oxidative stress also causes pathological polyploidization of hepatocytes [42]. Studies have shown that free fatty acids additionally induce lipotoxicity, which subsequently increases levels of nuclear factor kappa-light-chain-enhancer of activated B cells (NF-kβ) and those of tumor necrosis factor alpha (TNF-α), transforming growth factor beta (TGFβ-1), and interleukin-6 (IL-6) [43].

## 4. The Insulin Signaling Pathway

**Figure 2:**
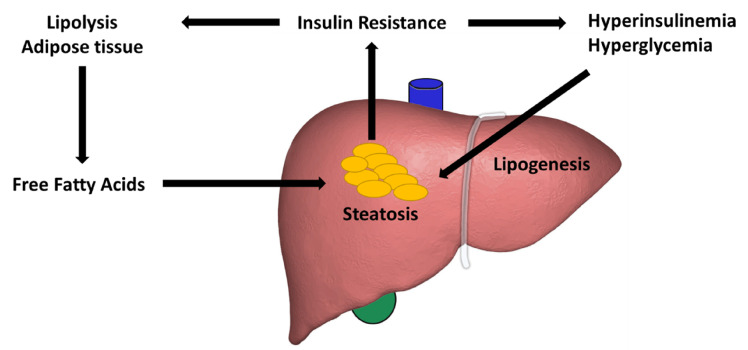
Role of insulin resistance in hepatic steatosis.

One of the most widely proposed pathways in the progression of NAFLD and hepatic fibrosis is the metabolic or insulin signaling pathway, which is associated with insulin resistance/hyperinsulinemia and hyperglycemia (Figure 2). Hyperinsulinemia is associated with T2D and obesity. These two conditions have been implicated closely in the progression of NAFLD to liver fibrosis. Other comorbid conditions include hyperlipidemia (69%), hypertriglyceridemia (41%), metabolic syndrome (43%), and hypertension (39%) [18].

Another relevant element affecting NAFLD is hyperglycemia, which stimulates insulin secretion and expands triglyceride synthesis by the liver, resulting in an increase in triglycerides in the blood and a buildup in the liver, leading to progressive liver injury and fibrosis [16].

Insulin operates by binding to tyrosine kinase receptors expressed at the plasma membrane of hepatocytes. There are two types of insulin receptors (IR) that come about via the alternative splicing of IR pre-mRNA: IR-A and IR-B, with the former being expressed in embryonic and fetal liver cells while the latter is solely expressed in adult hepatocytes [15]. Once insulin binds to IR-B, it causes the autophosphorylation of tyrosine kinase receptors and phosphorylation of its substrates. These, in turn, activate the phosphatidylinositol 3-kinase (PI3K)–AKT and Ras/mitogen-activated protein kinase (MAPK) pathways [22]. Insulin stimulation of AKT activity through 3-phosphoinositide-dependent protein kinase 1 (PDK-1) and mammalian target of rapamycin complex 2 (mTORC2)-dependent mechanisms leads to the inhibition of the transcription factor Forkhead box O 1 (FOXO1) [44,45].

However, as seen in mice models in insulin resistance states, high insulin levels have been unable to block transcription factor FOXO1 through AKT2-dependent phosphorylation. It is stipulated that FOXO1 activity correlates with lipogenesis, increased expression of IRS-2, and increased activation of AKT2. The upregulation of these receptors as well as activation of Wnt/β-catenin are thought to be involved in the development of liver fibrosis [41,46]. Ectopic expression of wild-type (WT) or mutant β-catenin on its own is not sufficient to induce HCC; however its expression helps accelerate the process of tumorigenesis [47].

## 5. The Role of Autophagy

Another essential part of metabolism is the process of autophagy and its involvement in malignant cell transformation. Autophagy is involved in homeostasis maintenance in cells by disposing of damaged organelles, as well as reducing intracellular lipids via fusion with lysosomes for degradation [48]. Autophagy has been increasingly investigated in NAFLD as recent studies have revealed that regulation of autophagy has therapeutic potential by decreasing liver inflammation and injury. Rat studies demonstrated lower autophagic potential in primary rat HCC cells when compared to normal cells. Autophagy-related genes (Atgs) have been discovered in yeast, which have mammalian homologs, such as *Beclin 1* (mammalian homolog of yeast *Atg6*) [48]. Mono-allelic deletion of Beclin 1 has been seen in various cancers, including liver, ovarian, breast, prostate, glioma and colon. The *PTEN* tumor-suppressor genes and the *AKT*, *RAS*, or *MYC* oncogenes have also been found to play a role in autophagy and tumor formation [48]. However, the actual mechanism by which autophagy functions in HCC is still unknown. During the early phase of tumor development, autophagy acts as a tumor suppressor and removes any damaged or mutated cells, thus maintaining stability. However, once a tumor is fully established, unbalanced autophagy cells act as tumor promotors, inducing tumor growth and further contributing to HCC cell survival [49].

## 6. Immunologic and Genetic Factors 

Another important mechanism involved in the progression of liver fibrosis involves an immunologic pathway. Two of the tumor-promoting cytokines, IL-6 and TNF, promote hepatic inflammation, as well as liver fat accumulation and activation of oncogenic transcription factor STAT3 eventually leading to the formation of HCC. It was found in mice models that loss or ablation of TNF and IL-6 prevents fat-induced liver injury and further development of HCC [50].

Natural killer (NK) cells are the immune cells that mostly reside within the liver and get activated by lipid antigens. It has been known that NK cells play one of the main roles in inflammation regulation; however, it is unclear whether NK cells exhibit pathogenic or suppressive function in obesity and NAFLD progression.

NK cells tend to accumulate in the liver due to the expression of NK cell ligands in murine models [51]. In these models, the expression of IL-15 has been shown to lead to NASH; however, NK cells have also been shown to play a protective role in progressive liver fibrosis. It has been suggested that this occurs through NK cell influence on macrophage polarization in the liver, by way of an NKp46-dependent mechanism, together with the destruction of hematopoietic stem cells via NKG2D-dependence [52]. Patients with NASH have been shown to have increasing expression of activating receptor NKG2D by circulating NK cells, which may be responsible for increased hematopoietic stem cell death in mouse models. On the other hand, lower NKG2D expression has been observed in circulating NK cells of NAFLD patients [53]. This downregulation of NKG2D within the context of NAFLD may give rise to more pro-fibrotic NK cells and lead to progressive liver fibrosis. However, this regulation may be in part due to metabolics.

Mice studies have revealed that CD1d^−/−^ mice that lack NKT cells were more prone to develop fatty liver and weight gain following a high-fat diet compared to their counterpart WT mice. These CD1d^−/−^ mice demonstrated increased stimulation of inflammatory genes in the liver seen in NAFLD. This suggests that NKT cells play a regulatory role that helps suppress and prevent the development of diet-induced obesity and hepatic inflammation by regulating crosstalk between metabolism and immune systems that maintains liver health and prevent progression to NAFLD [54].

In mice studies, NASH was induced after 2 months of treatment with methionine-choline deficient diets (MCD) [55]. This was demonstrated by the macrovascular steatosis and fibrosis in the liver of B6 mice. The number of NK cells seen in WT B6 mice was vastly increased in the liver; however, these were decreased in the spleen. These natural killer cells in the liver demonstrated elevated levels of activation induced by the increased expression of CD107a and the cytokines IFN-y, TGF-B, and IL-10. Decreased expression of Ki67 signified a decrease in the production of hepatic NK cells after MCD treatment. Soon after treatment with MCD, there was an elevated expression of CXCL which led to the recruitment of CXCR3 NK cells into the liver [55,56]. It was seen that reduction of NK cells during MCD-induced NASH caused a substantial surge in the infiltration of monocyte-derived macrophages. In all, intrahepatic NK cells seem to play a role in protection against fibrosis in NASH.

More recently, the role of mucosal-associated invariant T (MAIT) cells has been investigated in the context of chronic liver disease. MAIT cells have been associated with antimicrobial, immune regulatory, protective, and pathogenic roles [57]. As a subset of lymphocytes, they can be activated by cytokine stimulation in an antigen-independent manner, and they can eliminate infected or altered cells by releasing pro-apoptotic granzyme B and perforin [58]. While the direct role of MAIT cells in NAFLD is still unknown, Li et al. noted that MAIT cells were elevated in NAFLD and correlated with NAFLD activity score, while a protective effect was noted with macrophage polarization [59]. However, opposite effects have been found in MAIT cell-enriched mice, which demonstrated increased liver fibrosis and accumulation of hepatic fibrogenic cells compared to MAIT-deficient mice models [60]. Due to these initial and conflicting reports, more studies are needed to understand the regulation of MAIT cells and their role in progressive liver fibrosis.

Genetic modifiers have been shown to play a role in the pathogenesis of fatty liver disease and progressive fibrosis. One of the most well-characterized genes is patatin-like phospholipase domain-containing protein 3 (*PNPLA3*). PNLPA3 has been found to be directly associated with hepatic steatosis, steatohepatitis, elevated plasma liver enzyme levels, hepatic fibrosis, and cirrhosis [61,62,63,64]. *PNLPA3* is produced by the liver and has a role in retinol metabolism and hepatic inflammation [65].

In murine models, *PNLPA3* is upregulated in diets high in fats and carbohydrates, which creates an anabolic environment [66,67,68,69]. Mice deficient in *PNPLA3* on a high-fat diet have been shown to have reduced liver fat content. On the other hand, *PNPLA3* mediates the transfer of polyunsaturated fatty acids from triglycerides to phospholipids in hepatocytes. In other words, elevated *PNPLA3* protein levels lead to lipogenesis.

By contrast, deficient *PNPLA3* expression can potentially mitigate its negative effect on hepatic lipolysis [70]. However, some reports have indicated opposite effects which contradict the above reports. Altogether, the there is evidence to indicate that *PNPLA3* plays a significant role in steatosis and liver fibrosis; however, there are unknown confounding variables which may exert up- or downstream effects, which needs to be further studied.

## 7. Endocrine Pathway

The endocrine pathway is another mechanism that is involved in the development of NASH and NAFLD with progression to fibrosis by hormonal dysregulation. Endocrine hormones are responsible for lipid distribution and cell metabolism and dysfunction of these processes accelerates fat accumulation in the liver and metabolic liver disease. Multiple conditions, such as hypothyroidism and growth hormone deficiency have been linked to the progression of NASH. Obesity and hyperlipidemia are one of the main manifestations of a hypothyroid state, which leads to the development of NASH and eventually HCC. Polycystic ovarian syndrome is another endocrine condition associated with metabolic syndrome, insulin resistance, and the development of NASH [71].

**Figure 3:**
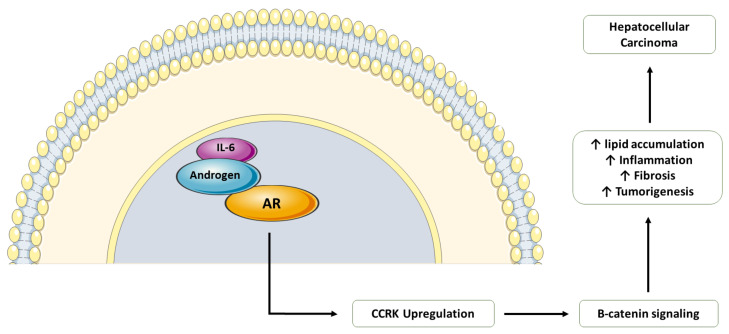
Role of androgens and estrogens in liver fibrosis and tumorigenesis. AR: androgen receptor, CCRK: cell cycle-related kinase.

As has been discussed above, obesity leads to the development of NAFLD and increases the risk of HCC, and this has been increasingly observed in males. An androgen receptor (AR)-driven oncogene called cell cycle-related kinase (CCRK) cooperates with obesity-induced pro-inflammatory signaling, leading to the development of NASH-related tumorigenesis of the liver (Figure 3). CCRK works by inducing STAT3-AR promoter and transcription upregulation that subsequently activates mTORC1/4E-BP1/S6K/SREBP1 cascades via GSK3β phosphorylation. STAT3-AR-CCRK-mTORC1 pathway components have been observed to be overexpressed in NASH-associated HCC [72]. Recent studies have revealed that ablation of CCRK by lentivirus in male mice that were fed with high-fat and high-carbohydrate diets prevents obesity-associated lipid accumulation and insulin resistance, as well as the development of HCC [73].

Lastly, recent genetic studies have further elucidated the role of AR in hepatocarcinogenesis and the relevance of increased HCC in males compared to females. Nagasue et al. looked at recurrence rates of HCC after resection [74]. The study revealed a strong association between androgen receptors and recurrence of intrahepatic HCC [74]. AR plays an important role in liver disease progression by the following signaling mechanism. CCRK transcription activation by ligand-bound AR induces cell cycle progression, the proliferation of hepatic cells, and malignant transformation. Out-of-place expression of CCRK in immortalized human liver cells activated β-catenin/T-cell factor signaling that has a similar role in cell cycle progression and tumor induction as CCRK. Thus, in studies in which knockdown of CCRK was attempted, decreased HCC cell growth was seen. However, this effect could be reversed by the activation of β-catenin/T-cell factor. Overexpression of all three receptors has been observed in human HCC tissue samples [75].

## 8. The Gut Microbiota

Gut microbiota has been found to promote NAFLD. Alterations to the intestinal microbiota or dysbiosis has been found to promote toxicity by altering bile metabolism, increasing intestinal permeability, induction of inflammasomes, increased energy extraction, altering choline metabolism, and stimulation of toxins [76].

Increased toxin production has been associated with dysbiosis, with examples including altered choline metabolism leading to toxic metabolites, such as trymethylamine, and direct stimulation of endogenous ethanol [76]. Altered microbiota has also been shown to affect lipid metabolism by parallel mechanisms, such as decreasing lipid breakdown by inhibiting B-oxidation and promoting lipid formation by stimulating lipoprotein lipase activity, short-chain fatty acid (SCFA) extraction, and enterohepatic circulation of bile acids [77]. Lastly, direct toxicity has been observed by obesogenic bacteria, leading to the formation of small intestine bacterial overgrowth (SIBO), bacterial translocation and subsequent whole-body inflammation by the stimulation of inflammasomes by bacterial endogenous products [77].

Despite studies linking dysbiosis and NAFLD, there is not any direct evidence to support alterations in gut microbiota and liver fibrosis [78]. It is also unclear if dysbiosis provokes or precedes NAFLD, NASH, or progressive liver fibrosis.

When compared with healthy controls, reports show a reduced microbial diversity correlated with obesity and NAFLD. Patients with higher-stage fibrosis or NASH with cirrhosis have significantly greater proportions of *Bacteroides* and *Ruminococcus*. A similar gut microbial pattern has been found in patients with type 2 diabetes compared to healthy controls [79]. The microbiome among patients with NAFLD varies widely, and differences in ethnicity, as well as environment, may have different effects on the progression of liver fibrosis [80]. Long-term, multiethnic studies are needed to understand the interplay between the microbiome and the rate of liver fibrosis in patients with NAFLD.

## 9. Biomarkers

Serum markers can be broken down into the pathological processes involved in NASH progression. Categories include inflammation, oxidation, adipokine signaling, and apoptosis/necrosis [81]. Inflammatory markers, such as IL-8, TGF-beta, IL-6, C-reactive protein, and ferritin have been associated with hepatic fibrosis given involvement in the recruitment of neutrophils and initiation of hepatocellular inflammation [82]. Ferritin, specifically, has been found to be a good predictor of NASH, more so when evaluated in conjunction with other factors, such as AST levels and BMI. The combination of these factors with ferritin increases the diagnostic accuracy of this commonly tested acute-phase reactant [83]. These markers are available commercially and are commonly used in clinical practice; however, they are limited by their diagnostic value given their non-specificity as they can be affected by systemic inflammation.

Oxidation damage, peroxidation of lipids, and microbial activity can increase caspase activity in the setting of worsening mitochondrial permeability. The effect is steatosis and tissue death by apoptosis and necrosis. Cytokeratin (CK) 18 is a filament protein in hepatocytes that is cleaved by caspases during the initiation of cell death. It is a well-validated blood biomarker for apoptosis that is used commercially to correlate histological improvement over time. FAS is also released by caspases and has been associated with the extensive apoptosis pathway in hepatocytes [84]. The accuracy of these markers is limited if used in isolation and is associated with low sensitivity.

Oxidative stress, marked by lipid oxidation products, has been associated with NASH. Specifically, products of arachidonic acid oxidation and linoleic acid oxidation have had the greatest evidence of correlation from amongst the oxidation products [85]. These markers have been incorporated in diagnostic panels, such as the oxNASH score [86], but are limited in clinical usability given the cost of the equipment needed to measure these end by-products.

Hormones have been found to correlate with NASH pathogenesis, such as adiponectin, leptin, and resistin. Adiponectin is an adipose tissue-made protein negatively associated with insulin resistance, diabetes, and dyslipidemia due to its impact on fatty acid metabolism and insulin-receptor function [87]. Lower levels of adiponectin have been found to be associated with fibrosis based on a 2011 meta-analysis [88]. Leptin is another protein hormone involved with food intake regulation and was found to be directly correlated with steatosis in obese patients. Unfortunately, these adipokines are mostly validated only in obese populations and are poorly specific due to their reflection of general visceral adiposity [89].

Abnormal liver function tests, and specifically elevations in AST and ALT, are the most used markers for hepatic disease. They are non-specific indicators of hepatic fibrosis when ALT is larger than AST, compared to the opposite association in alcohol-related cirrhosis. The ratio of ALT/AST has been found to be a poor indicator of fibrosis with an accuracy of 0.66–0.74. The elevations in liver enzymes, however, do not correlate with the degree of fibrosis and are considered poor predictors of NASH when used in isolation [90,91,92,93,94].

Liver function tests (LFTs) have been incorporated in algorithms, such as Fibrosis-4 Index (FIB-4) to improve the accuracy of detecting fibrosis [95]. FIB-4 is an algorithm that was initially used to stage liver disease in patients with hepatitis C, but, its use has been expanded to detect for NASH by incorporating age and platelet level, in conjunction with LFTs [91]. However, a key disadvantage is its propensity to overestimate fibrosis in alcohol users, due to its heavy reliance on AST [95]. Nonetheless, FIB-4 has been found to increase sensitivity of fibrosis detection and decrease the needs for liver biopsies, especially when combined with imaging-based biomarkers, such as liver stiffness measurement (LSM) detected by acoustic resonance form impulse (ARFI) and vibration controlled transient elastography (VCTE) [96].

While image modalities perform poorly in detection of NASH when used independently, especially in obese patients and in the presence of type 2 diabetes, there has been significant progress and success in their use in-conjugation with the aforementioned algorithms, such as FIB-4, to act as a surrogate for the detection of fibrosis [97]. VCTE obtains the median liver stiffness measurement in kilopascals, while ARFI quantitatively measures liver stiffness using shear wave speed in meters per second, with ARFI appearing to have a stronger ability to estimate fibrosis compared to VCTE [97].

Molecular biomarkers, such as metalloproteinases and extracellular matrix by-products, have been found to promote strong predictions of risk for developing fibrosis, to confirm fibrosis, and to monitor fibrosis progression [98]. A key by-product includes hyaluronic acid (HA), an extracellular matrix polymer released by hepatic stellate cells as a result of activation in the setting of chronic liver disease [98]. HA has been incorporated into algorithms, such as Enhanced Liver Fibrosis Score and HepaScore; however, limitations include the need to develop age and ethnic-specific differences in cut-off scores [98].

Liver biopsy continues to be the gold standard for the diagnosis of NAFLD and liver fibrosis. However, given the invasive nature of the procedure, patented algorithms have been designed by incorporating multiple demographic and metabolic parameters, in order to provide a non-invasive alternative. The NASHTest is one such panel that includes some markers, such as AST, ALT, and total bilirubin, and was found to have an area under the ROC curves of 0.69–0.83 [99]. In 2008, Younossi et al. created two algorithms, the first incorporated apoptosis markers and adipokines, such as CK-18 and resistin and was found to have an area under the ROC curves of 0.73–0.91 for NASH. The second panel, called The NASH Diagnostic panel included demographics as well as CK18 to create a diagnostic tool with an average accuracy of 0.81 [100]. While these biomarker panels offer hope for a safer, non-invasive alternative to liver biopsies, the aforementioned algorithms used small populations that consisted of obese patients. External validity is inadequate unless more studies are performed using larger and more diverse populations that include non-obese subjects.

## 10. Conclusions

The growing incidence of NASH represents a public health threat with significant cost to healthcare systems. Our review shows several pathways by which NASH progresses to liver fibrosis. These mechanisms are being further elucidated but represent possible avenues for biomedical intervention. Furthermore, it underscores the complexity of this disease and the effects of cellular, genetic, immunologic, metabolic, and endocrine contributions. While no FDA-approved medications are available, there are phase III clinical candidates being explored that may hold promise for patients with NASH. Further work is needed if we are to stem this growing tide and develop methods for decreasing steatosis, NASH, fibrosis, and HCC.

## Data Availability

Not applicable.

## References

[1] Wree A., Broderick L., Canbay A., Hoffman H.M., Feldstein A.E. (2013). From NAFLD to NASH to cirrhosis—New insights into disease mechanisms. Nat. Rev. Gastroenterol. Hepatol..

[2] Wanless I.R., Lentz J.S. (1990). Fatty liver hepatitis (steatohepatitis) and obesity: An autopsy study with analysis of risk factors. Hepatology.

[3] Gutiérrez-Cuevas J., Santos A., Armendariz-Borunda J. (2021). Pathophysiological molecular mechanisms of obesity: A link between MAFLD and NASH with cardiovascular diseases. Int. J. Mol. Sci..

[4] Younossi Z.M., Golabi P., de Avila L., Paik J.M., Srishord M., Fukui N., Qiu Y., Burns L., Afendy A., Nader F. (2019). The global epidemiology of NAFLD and NASH in patients with type 2 diabetes: A systematic review and meta-analysis. J. Hepatol..

[5] Pan J.J., Fallon M.B. (2014). Gender and racial differences in nonalcoholic fatty liver disease. World J. Hepatol..

[6] Clark J.M., Brancati F.L., Diehl A.M. (2003). The prevalence and etiology of elevated aminotransferase levels in the United States. Am. J. Gastroenterol..

[7] Ioannou G.N., Boyko E.J., Lee S.P. (2006). The prevalence and predictors of elevated serum aminotransferase activity in the United States in 1999–2002. Am. J. Gastroenterol..

[8] Kanwal F., Kramer J.R., Duan Z., Yu X., White D., El-Serag H.B. (2016). Trends in the Burden of Nonalcoholic Fatty Liver Disease in a United States Cohort of Veterans. Clin. Gastroenterol. Hepatol..

[9] Wong M., Huang J., George J., Huang J., Leung C., Eslam M., Chan H., Ng S.C. (2019). The changing epidemiology of liver diseases in the Asia-Pacific region. Nat. Rev. Gastroenterol. Hepatol..

[10] Mitra S., De A., Chowdhury A. (2020). Epidemiology of non-alcoholic and alcoholic fatty liver diseases. Transl. Gastroenterol. Hepatol..

[11] Schneider A.L., Lazo M., Selvin E., Clark J.M. (2014). Racial differences in nonalcoholic fatty liver disease in the U.S. population. Obesity.

[12] Romeo S., Kozlitina J., Xing C., Pertsemlidis A., Cox D., Pennacchio L.A., Boerwinkle E., Cohen J.C., Hobbs H.H. (2008). Genetic variation in PNPLA3 confers susceptibility to nonalcoholic fatty liver disease. Nat. Genet..

[13] Ramai D., Tai W., Rivera M., Facciorusso A., Tartaglia N., Pacilli M., Sacco R. (2021). Natural Progression of Non-Alcoholic Steatohepatitis to Hepatocellular Carcinoma. Biomedicines.

[14] Lazarus J.V., Mark H.E., Anstee Q.M., Arab J.P., Batterham R.L., Castera L., Cortez-Pinto H., Crespo J., Cusi K., Dirac M.A. (2021). Advancing the global public health agenda for NAFLD: A consensus statement. Nat. Rev. Gastroenterol. Hepatol..

[15] Dietrich P., Hellerbrand C. (2014). Non-alcoholic fatty liver disease, obesity and the metabolic syndrome. Best Pract. Res. Clin. Gastroenterol..

[16] Qureshi K., Abrams G.A. (2007). Metabolic liver disease of obesity and role of adipose tissue in the pathogenesis of nonalcoholic fatty liver disease. World J. Gastroenterol..

[17] Van der Poorten D., Milner K.-L., Hui J., Hodge A., Trenell M.I., Kench J.G., London R., Peduto T., Chisholm D.J., George J. (2008). Visceral fat: A key mediator of steatohepatitis in metabolic liver disease. Hepatology.

[18] Petta S., Amato M.C., Di Marco V., Cammà C., Pizzolanti G., Barcellona M.R., Cabibi D., Galluzzo A., Sinagra D., Giordano C. (2012). Visceral adiposity index is associated with significant fibrosis in patients with non-alcoholic fatty liver disease. Aliment. Pharmacol. Ther..

[19] Facciorusso A. (2013). The influence of diabetes in the pathogenesis and the clinical course of hepatocellular carcinoma: Recent findings and new perspectives. Curr. Diabetes. Rev..

[20] Garg K., Brackett S., Hirsch I., Garg S. (2020). NAFLD/NASH and Diabetes. Diabetes Technol. Ther..

[21] Pang Y., Kartsonaki C., Turnbull I., Guo Y., Clarke R., Chen Y., Bragg F., Yang L., Bian Z., Millwood I. (2018). Diabetes, Plasma Glucose, and Incidence of Fatty Liver, Cirrhosis, and Liver Cancer: A Prospective Study of 0.5 Million People. Hepatology.

[22] Lonardo A., Nascimbeni F., Mantovani A., Targher G. (2018). Hypertension, diabetes, atherosclerosis and NASH: Cause or consequence?. J. Hepatol..

[23] Maciejewska-Markiewicz D., Stachowska E., Hawryłkowicz V., Stachowska L., Prowans P. (2021). The role of resolvins, protectins and marensins in non-alcoholic fatty liver disease (NAFLD). Biomolecules.

[24] Schuppan D., Surabattula R., Wang X.Y. (2018). Determinants of fibrosis progression and regression in NASH. J. Hepatol..

[25] Charlton M., Krishnan A., Viker K., Sanderson S., Cazanave S., McConico A., Masuoko H., Gores G. (2011). Fast food diet mouse: Novel small animal model of NASH with ballooning, pro413 gressive fibrosis, and high physiological fidelity to the human condition. Am. J. Physiol. Gastrointest. Liver Physiol..

[26] Neuschwander-Tetri B.A. (2009). Lifestyle modification as the primary treatment of NASH. Clin. Liver. Dis..

[27] Guerrero R., Vega G.L., Grundy S.M., Browning J.D. (2009). Ethnic differences in hepatic steatosis: An insulin resistance paradox?. Hepatology.

[28] Dongiovanni P., Anstee Q.M., Valenti L. (2013). Genetic predisposition in NAFLD and NASH: Impact on severity of liver disease and response to treatment. Curr. Pharm. Des..

[29] Dludla P.V., Nkambule B.B., Mazibuko-Mbeje S.E., Nyambuya T.M., Marcheggiani F., Cirilli I., Ziqubu K., Shabalala S.C., Johnson R., Louw J. (2020). N-Acetyl Cysteine Targets Hepatic Lipid Accumulation to Curb Oxidative Stress and Inflammation in NAFLD: A Comprehensive Analysis of the Literature. Antioxidants.

[30] Pierantonelli I., Svegliati-Baroni G. (2019). Nonalcoholic Fatty Liver Disease. Transplantation.

[31] Lara-Castro C., Garvey W. (2008). Intracellular Lipid Accumulation in Liver and Muscle and the Insulin Resistance Syndrome. Endocrinol. Metabol. Clin. North. Am..

[32] Arner P. (2003). The adipocyte in insulin resistance: Key molecules and the impact of the thiazolidinediones. Trends Endocrinol. Metab..

[33] Samuel V.T., Shulman G.I. (2012). Mechanisms for insulin resistance: Common threads and missing links. Cells.

[34] Aboubakr A., Stroud A., Kumar S., Newberry C. (2021). Dietary approaches for management of non-alcoholic fatty liver disease: A clinician’s guide. Curr. Gastroenterol. Rep..

[35] Al-Busafi S.A., Bhat M., Wong P., Ghali P., Deschenes M. (2012). Antioxidant Therapy in Nonalcoholic Steatohepatitis. Hepat. Res. Treat..

[36] Browning J.D., Horton J.D. (2004). Molecular mediators of hepatic steatosis and liver injury. J. Clin. Investig..

[37] Brunt E.M., Kleiner D.E., Wilson L.A., Belt P., Neuschwander-Tetri B.A. (2010). For the NASH Clinical Research Network (CRN) Nonalcoholic fatty liver disease (NAFLD) activity score and the histopathologic diagnosis in NAFLD: Distinct clinicopathologic meanings. Hepatology.

[38] George D., Goldwurm S., Macdonald G.A., Cowley L.L., Walker N.I., Ward P.J., Jazwinska E.C., Powell L.W. (1998). Increased hepatic iron concentration in nonalcoholic steatohepatitis is associated with increased fibrosis. Gastroenterology.

[39] Zangar R.C., Novak R.F. (1997). Effects of Fatty Acids and Ketone Bodies on Cytochromes P450 2B, 4A, and 2E1 Expression in Primary Cultured Rat Hepatocytes. Arch. Biochem. Biophys..

[40] Ockner R.K., Kaikus R.M., Bass N.M. (1993). Fatty-acid metabolism and the pathogenesis of hepatocellular carcinoma: Review and hypothesis. Hepatology.

[41] Ore A., Akinloye O.A. (2019). Oxidative stress and antioxidant biomarkers in clinical and experimental models of non-alcoholic fatty liver disease. Medicina.

[42] Gentric G., Maillet V., Paradis V., Couton D., L’Hermitte A., Panasyuk G., Fromenty B., Celton-Morizur S., Desdouets C. (2015). Oxidative stress promotes pathologic polyploidization in nonalcoholic fatty liver disease. J. Clin. Investig..

[43] Feldstein A.E., Werneburg N.W., Canbay A., Guicciardi M.E., Bronk S.F., Rydzewski R., Burgart L.J., Gores G.J. (2004). Free fatty acids promote hepatic lipotoxicity by stimulating TNF-alpha expression via a lysosomal pathway. Hepatology.

[44] Boucher J., Kleinridders A., Kahn C.R. (2014). Insulin receptor signaling in normal and insulin-resistant states. Cold Spring Harb. Perspect. Biol..

[45] Lu M., Wan M., Leavens K.F., Chu Q., Monks B.R., Fernandez S., Ahima R.S., Ueki K., Kahn C.R., Birnbaum M.J. (2012). Insulin regulates liver metabolism in vivo in the absence of hepatic Akt and Foxo1. Nat. Med..

[46] Russell J.O., Monga S.P. (2018). Wnt/β-Catenin Signaling in Liver Development, Homeostasis, and Pathobiology. Annu. Rev. Pathol..

[47] Elsayed H.R.H., El-Nablaway M., Khattab B.A., Sherif R.N., Elkashef W.F., Abdalla A.M., El Nashar E.M., Abd-Elmonem M.M., El-Gamal R. (2021). Independent of Calorie Intake, Short-term Alternate-day Fasting Alleviates NASH, With Modulation of Markers of Lipogenesis, Autophagy, Apoptosis, and Inflammation in Rats. J. Histochem. Cytochem..

[48] Liu L., Liao J.Z., He X.X., Li P.Y. (2017). The role of autophagy in hepatocellular carcinoma: Friend or foe. Oncotarget.

[49] Mao Y., Yu F., Wang J., Guo C., Fan X. (2016). Autophagy: A new target for nonalcoholic fatty liver disease therapy. Hepat. Med..

[50] Park E.J., Lee J.H., Yu G.Y., He G., Ali S.R., Holzer R.G., Karin M. (2010). Dietary and genetic obesity promote liver inflammation and tumorigenesis by enhancing IL-6 and TNF expression. Cell.

[51] Highton A.J., Schuster I.S., Degli-Esposti M.A., Altfeld M. (2021). The role of natural killer cells in liver inflammation. Semin. Immunopathol..

[52] Tosello-Trampont A.C., Krueger P., Narayanan S., Landes S.G., Leitinger N., Hahn Y.S. (2016). NKp46(+) natural killer cells attenuate metabolism-induced hepatic fibrosis by regulating macrophage activation in mice. Hepatology.

[53] Martínez-Chantar M.L., Delgado T.C., Beraza N. (2021). Revisiting the Role of Natural Killer Cells in Non-Alcoholic Fatty Liver Disease. Front. Immunol..

[54] Martin-Murphy B.V., You Q., Wang H., Becky A., Reilly T.P., Friedman J.E., Ju C. (2014). Mice lacking natural killer T cells are more susceptible to metabolic alterations following high fat diet feeding. PLoS ONE.

[55] Fan Y., Zhang W., Wei H., Sun R., Tian Z., Chen Y. (2020). Hepatic NK cells attenuate fibrosis progression of non-alcoholic steatohepatitis in dependent of CXCL10-mediated recruitment. Liver Int..

[56] Zheng X., Zeng W., Gai X., Xu Q., Li C., Liang Z., Liu Q. (2013). Role of the Hedgehog pathway in hepatocellular carcinoma. Oncol. Rep..

[57] Czaja A.J. (2021). Incorporating mucosal-associated invariant T cells into the pathogenesis of chronic liver disease. World J. Gastroenterol..

[58] Bolte F.J., Rehermann B. (2018). Mucosal-Associated Invariant T Cells in Chronic Inflammatory Liver Disease. Liver. Dis..

[59] Li Y., Huang B., Jiang X., Chen W., Zhang J., Wei Y., Chen Y., Lian M., Bian Z., Miao Q. (2018). Mucosal-Associated Invariant T Cells Improve Nonalcoholic Fatty Liver Disease Through Regulating Macrophage Polarization. Front. Immunol..

[60] Hegde P., Weiss E., Paradis V., Wan J., Mabire M., Sukriti S., Rautou P.E., Albuquerque M., Picq O., Gupta A.C. (2018). Mucosal-associated invariant T cells are a profibrogenic immune cell population in the liver. Nat. Commun..

[61] Singal A.G., Manjunath H., Yopp A.C., Beg M.S., Marrero J.A., Gopal P., Waljee A.K. (2014). The Effect of PNPLA3 on Fibrosis Progression and Development of Hepatocellular Carcinoma: A Meta-analysis. Am. J. Gastroenterol..

[62] Sookoian S., Pirola C.J. (2011). Meta-analysis of the influence of I148M variant of patatin-like phospholipase domain containing 3 gene (PNPLA3) on the susceptibility and histological severity of nonalcoholic fatty liver disease. Hepatology.

[63] Bruschi F., Tardelli M., Claudel T., Trauner M. (2017). PNPLA3 expression and its impact on the liver: Current perspectives. Hepatic Med. Evid. Res..

[64] Pingitore P., Romeo S. (2019). The role of PNPLA3 in health and disease. Biochim. Biophys. Acta Mol. Cell Biol. Lipids.

[65] Baulande S., Lasnier F., Lucas M., Pairault J. (2001). Adiponutrin, a Transmembrane Protein Corresponding to a Novel Dietary- and Obesity-linked mRNA Specifically Expressed in the Adipose Lineage. J. Biol. Chem..

[66] Hao L., Ito K., Huang K.H., Sae-tan S., Lambert J.D., Ross A.C. (2014). Shifts in dietary carbohydrate-lipid exposure regulate expression of the non-alcoholic fatty liver disease-associated gene PNPLA3/adiponutrin in mouse liver and HepG2 human liver cells. Metabolism.

[67] Moldes M., Beauregard G., Faraj M., Peretti N., Ducluzeau P.-H., Laville M., Rabasa-Lhoret R., Vidal H., Clément K. (2006). Adiponutrin gene is regulated by insulin and glucose in human adipose tissue. Eur. J. Endocrinol..

[68] Huang Y., He S., Li J.Z., Seo Y.-K., Osborne T.F., Cohen J.C., Hobbs H.H. (2010). A feed-forward loop amplifies nutritional regulation of PNPLA3. Proc. Natl. Acad. Sci. USA.

[69] Dubuquoy C., Robichon C., Lasnier F., Langlois C., Dugail I., Foufelle F., Girard J., Burnol A.-F., Postic C., Moldes M. (2011). Distinct regulation of adiponutrin/PNPLA3 gene expression by the transcription factors ChREBP and SREBP1c in mouse and human hepatocytes. J. Hepatol..

[70] Perttilä J., Huaman-Samanez C., Caron S., Tanhuanpää K., Staels B., Yki-Järvinen H., Olkkonen V.M. (2012). PNPLA3 is regulated by glucose in human hepatocytes, and its I148M mutant slows down triglyceride hydrolysis. Am. J. Physiol. Endocrinol. Metab..

[71] Loria P., Carulli L., Bertolotti M., Lonardo A. (2009). Endocrine and liver interaction: The role of endocrine pathways in NASH. Nat. Rev. Gastroenterol. Hepatol..

[72] Sun H., Yang W., Tian Y., Zeng X., Zhou J., Mok M.T., Cheng A.S. (2018). An inflammatory-CCRK circuitry drives mTORC1-dependent metabolic and immunosuppressive reprogramming in obesity-associated hepatocellular carcinoma. Nat. Commun..

[73] Feng H., Yu Z., Tian Y., Lee Y.Y., Li M.S., Go Cheng M.Y., Cheung Y.S., Lai P.B., Chan A.M., To K.F. (2015). A CCRK-EZH2 epigenetic circuitry drives hepatocarcinogenesis and associates with tumor recurrence and poor survival of patients. J. Hepatol..

[74] Nagasue N., Yu L., Yukaya H., Kohno H., Nakamura T. (1995). Androgen and oestrogen receptors in hepatocellular carcinoma and surrounding liver parenchyma: Impact on intrahepatic recurrence after hepatic resection. Br. J. Surg..

[75] Awuah P.K., Monga S.P. (2012). Cell cycle–related kinase links androgen receptor and β-catenin signaling in hepatocellular carcinoma: Why are men at a loss?. Hepatology.

[76] Arslan N. (2014). Obesity, fatty liver disease and intestinal microbiota. World J. Gastroenterol..

[77] Machado M.V., Cortez-Pinto H. (2012). Gut microbiota and nonalcoholic fatty liver disease. Ann. Hepatol..

[78] Albhaisi S.A.M., Bajaj J.S., Sanyal A.J. (2019). Role of gut microbiota in liver disease. Am. J. Physiol. Gastrointest. Liver. Physiol..

[79] Bajaj J.S., Betrapally N.S., Hylemon P.B., Thacker L.R., Daita K., Kang D.J., White M.B., Unser A.B., Fagan A., Gavis E.A. (2015). Gut microbiota alterations can predict hospitalizations in cirrhosis independent of diabetes mellitus. Sci. Rep..

[80] Albhaisi S.A.M., Bajaj J.S. (2021). The Influence of the Microbiome on NAFLD and NASH. Clin. Liver. Dis..

[81] Neuman M.G., Cohen L.B., Nanau R.M. (2014). Biomarkers in nonalcoholic fatty liver disease. Can. J. Gastroenterol. Hepatol..

[82] Kwok R., Tse Y.K., Wong G.L., Ha Y., Lee A.U., Ngu M.C., Chan H.L., Wong V.W. (2014). Systematic review with meta-analysis: Non-invasive assessment of non-alcoholic fatty liver disease--the role of transient elastography and plasma cytokeratin-18 fragments. Aliment. Pharmacol. Ther..

[83] Manousou P., Kalambokis G., Grillo F., Watkins J., Xirouchakis E., Pleguezuelo M., Leandro G., Arvaniti V., Germani G., Patch D. (2011). Serum ferritin is a discriminant marker for both fibrosis and inflammation in histologically proven non-alcoholic fatty liver disease patients. Liver Int..

[84] Roskams T., Yang S.Q., Koteish A., Durnez A., DeVos R., Huang X., Achten R., Verslype C., Diehl A.M. (2003). Oxidative stress and oval cell accumulation in mice and humans with alcoholic and nonalcoholic fatty liver disease. Am. J. Pathol..

[85] Puri P., Wiest M.M., Cheung O., Mirshahi F., Sargeant C., Min H.K., Contos M.J., Sterling R.K., Fuchs M., Zhou H. (2009). The plasma lipidomic signature of nonalcoholic steatohepatitis. Hepatology.

[86] Lkhouri N., Berk M., Yerian L., Lopez R., Chung Y.M., Zhang R., McIntyre T.M., Feldstein A.E., Hazen S.L. (2014). OxNASH score correlates with histologic features and severity of nonalcoholic fatty liver disease. Dig. Dis. Sci..

[87] Armutcu F., Akyol S., Ucar F., Erdogan S., Akyol O. (2013). Markers in nonalcoholic steatohepatitis. Adv. Clin. Chem..

[88] Polyzos S.A., Toulis K.A., Goulis D.G., Zavos C., Kountouras J. (2011). Serum total adiponectin in nonalcoholic fatty liver disease: A systematic review and meta-analysis. Metabolism.

[89] Ozcelik F., Yuksel C., Arslan E., Genc S., Omer B., Serdar M.A. (2013). Relationship between visceral adipose tissue and adiponectin, inflammatory markers and thyroid hormones in obese males with hepatosteatosis and insulin resistance. Arch. Med. Res..

[90] Sheth S.G., Flamm S.L., Gordon F.D., Chopra S. (1998). AST/ALT ratio predicts cirrhosis in patients with chronic hepatitis C virus infection. Am. J. Gastroenterol..

[91] Facciorusso A., Del Prete V., Turco A., Buccino R.V., Nacchiero M.C., Muscatiello N. (2018). Long-term liver stiffness assessment in hepatitis C virus patients undergoing antiviral therapy: Results from a 5-year cohort study. J. Gastroenterol. Hepatol..

[92] Facciorusso A., Garcia Perdomo H.A., Muscatiello N., Buccino R.V., Wong V.W., Singh S. (2018). Systematic review with meta-analysis: Change in liver stiffness during anti-viral therapy in patients with hepatitis B. Dig. Liver Dis..

[93] Singh S., Facciorusso A., Loomba R., Falck-Ytter Y.T. (2018). Magnitude and Kinetics of Decrease in Liver Stiffness After Antiviral Therapy in Patients with Chronic Hepatitis C: A Systematic Review and Meta-analysis. Clin. Gastroenterol. Hepatol..

[94] Bellanti F., Villani R., Tamborra R., Blonda M., Iannelli G., di Bello G., Facciorusso A., Poli G., Iuliano L., Avolio C. (2018). Synergistic interaction of fatty acids and oxysterols impairs mitochondrial function and limits liver adaptation during nafld progression. Redox Biol..

[95] Sterling R.K., Lissen E., Clumeck N., Sola R., Correa M.C., Montaner J.S., Sulkowski M., Torriani F.J., Dieterich D.T., Thomas D.L. (2006). APRICOT Clinical Investigators. Development of a simple noninvasive index to predict significant fibrosis in patients with HIV/HCV coinfection. Hepatology.

[96] Mózes F.E., Lee J.A., Selvaraj E.A., Jayaswal A., Trauner M., Boursier J., Fournier C., Staufer K., Stauber R.E., Bugianesi E. (2021). Diagnostic accuracy of non-invasive tests for advanced fibrosis in patients with NAFLD: An individual patient data meta-analysis. Gut.

[97] Loomba R. (2018). Role of imaging-based biomarkers in NAFLD: Recent advances in clinical application and future research directions. J. Hepatol..

[98] Neuman M.G., Cohen L.B., Nanau R.M. (2016). Hyaluronic acid as a non-invasive biomarker of liver fibrosis. Clin. Biochem..

[99] Poynard T., Ratziu V., Charlotte F., Messous D., Munteanu M., Imbert-Bismut F., Massard J., Bonyhay L., Tahiri M., Thabut D. (2006). Diagnostic value of biochemical markers (NashTest) for the prediction of non alcoholo steato hepatitis in patients with non-alcoholic fatty liver disease. BMC Gastroenterol..

[100] Younossi Z.M., Jarrar M., Nugen C., Randhawa M., Afendy M., Stepanova M., Rafiq N., Goodman Z., Chandhoke V., Baranova A. (2008). A novel diagnostic biomarker panel for obesity-related nonalcoholic steatohepatitis (NASH). Obes. Surg..

